# A Workflow for Selection of Single Nucleotide Polymorphic Markers for Studying of Genetics of Ischemic Stroke Outcomes

**DOI:** 10.3390/genes12030328

**Published:** 2021-02-25

**Authors:** Gennady Khvorykh, Andrey Khrunin, Ivan Filippenkov, Vasily Stavchansky, Lyudmila Dergunova, Svetlana Limborska

**Affiliations:** Institute of Molecular Genetics of National Research Centre, “Kurchatov Institute”, 123182 Moscow, Russia; khrunin@img.ras.ru (A.K.); filippenkov@img.ras.ru (I.F.); bacbac@yandex.ru (V.S.); dergunov@img.ras.ru (L.D.); limbor@img.ras.ru (S.L.)

**Keywords:** single nucleotide polymorphisms, models of brain ischemia, human orthologues, ischemic stroke

## Abstract

In this paper we propose a workflow for studying the genetic architecture of ischemic stroke outcomes. It develops further the candidate gene approach. The workflow is based on the animal model of brain ischemia, comparative genomics, human genomic variations, and algorithms of selection of tagging single nucleotide polymorphisms (tagSNPs) in genes which expression was changed after ischemic stroke. The workflow starts from a set of rat genes that changed their expression in response to brain ischemia and results in a set of tagSNPs, which represent other SNPs in the human genes analyzed and influenced on their expression as well.

## 1. Introduction

The ischemic stroke (IS) is a multifactorial disease, where the genetic factors contribute substantially [1]. The same seems to be true for outcomes after IS. However, their associations with the particular genetic factors are poorly known and require further investigation [2,3]. There are two main approaches to identify the genes involved in the development of complex traits: candidate gene approach and genome-wide association (GWA) study (GWAS) [4]. Both were extensively applied to study the genetic bases of IS and resulted in revealing several tens of genes involved in stroke development and risk [5]. In contrast, only few GWA studies have been published on outcomes after IS [6,7]. Therefore, the real genetic control of them remains a black box and the full list of the risk (prognostic) loci is yet to be identified. In this paper we describe an approach to explore the genetic bases of variability in IS outcomes. 

GWAS does not require the prior knowledge on the importance of the specific functional features of the trait under consideration. At the same time, it is less precise in revealing causal loci (genes) generally located in particular chromosomal regions that can contain no genes or alternatively be abundant with them [8]. The usability of a gene-based approach was mainly restricted by the incompleteness of knowledge about the biology of the phenotypes studied. To break the information bottleneck, several strategies extending the candidate gene approach were proposed [4]. They were based on linkage information in a chromosomal segment, methods of comparative genomics, and gene expression at different stages. There were also the approaches that combine two or more strategies together. One such method is the digital candidate gene approach (DigiCGA), which extract, filter, and analyze the resources on the web available publicly [9]. The method we propose incorporates the best strategies of the mentioned above approaches and puts them in a form of a workflow.

The idea of this research originates from the models of brain ischemia in laboratory animals that were developed to understand the biological processes underlying cerebral ischemic injury [10]. Studies of rat and mouse genomes showed that most part of human disease genes (99.5%) had orthologues in rodents [11]. Furthermore, comparison of conservation rates of rodent orthologues associated with different types of diseases demonstrated that gene set related to neurological conditions evolved slowly. Together that suggested the rodent models of human neurological diseases to be appropriate representations of the disease processes in humans. Many of the results obtained in model experiments were subsequently confirmed (correlated) in corresponding GWA studies in humans, including those assessed with outcomes after IS [6]. Although there is no animal model that could cover all aspects of human ischemic stroke [12], one of such models—the transient middle cerebral artery occlusion (tMCAO)—is quite promising and actively tested for the development of neuroprotective therapeutic approaches. It is based on temporal artery occlusion and subsequent restoration of blood flow. According to Howells, such model was used in 42.2% of 2582 neuroprotection experiments. The occlusion with subsequent restoration of blood flow can influence the functioning of different genes. Recently, Dergunova et al. identified a list of rat genes that substantially changed their expression in brain in the response to tMCAO [13]. We propose to explore the genomic variations in human orthologues of these genes for searching the genomic markers of IS outcome. Below, we describe in the details the workflow that starts from the list of the rat genes and leads to a set of tagging SNPs (tagSNP) that can be used in case–control studies with the conventional TaqMan real-time PCR assays. 

## 2. Materials and Methods

**Figure 1:**
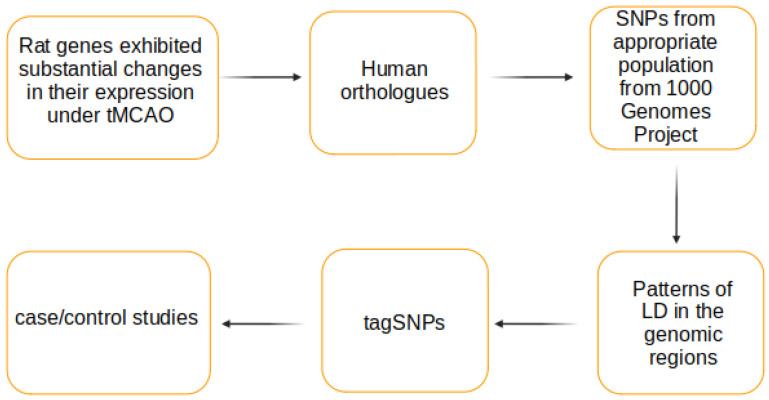
The workflow to identify the tagging SNPs for studying the ischemic stroke outcomes.

The main steps of the workflow proposed are shown in Figure 1. In the beginning, there are rat genes with expression level evaluated at 24 h after tMCAO [13]. Twenty-four of them demonstrated the most significant changes in expression level (change in expression >6-fold and *p*-value < 0.01) and were chosen for further analysis.

The human orthologues of the rat genes were comparatively identified by querying several resources: Ensembl [14], PANTHER 8.0 [15], PhylomeDB 4 [16], and MetaPhOrs [17]. The data from the database Ensembl Genes 97 were retrieved with BioMart by accessing it with web-based interface [18]. 

The next step was the identification of SNPs within the human genes, including their 5’ and 3’ flanking regions of 5000 bp length. To be relevant to the SNP frequencies in the potential case–control study, the genotypic data should be taken from an appropriate population [19]. To choose such a population, the collection of population samples of 1000 Genomes Project was used. The project comprises one the most comprehensively characterized set of populations with detailed history about each of them [20]. For our purposes we selected CEU population because its genotype data had been shown to be appropriate for selection of loci to assess genetic variability in the most European populations, including those living in Russia [21,22,23,24]. We extracted the required set of SNPs from the bulk of CEU genotype data using VCFtools (0.1.15) [25]. To capture the most common genetic variants, the SNPs with minor allele frequency (MAF) higher than 10% were considered.

Then, we explored the associations between the alleles of selected loci using the correlation coefficient r2 and revealed patterns of linkage disequilibrium (LD) in each of the region considered. To do this, we applied the CLUSTAG tool [26], Tagger instrument [27] implemented in Haploview 4.2 tool [28], and gpart R package (version 1.2.0) [29] using default parameters.

The input files were generated from vcf files obtained in the previous step with the custom scripts. All of the tools were able to reveal patterns of LD (LD blocks) using distinct algorithms but only CLUSTAG and Haploview allowed to compute tagSNPs which represented the groups of highly correlated SNPs in a chromosomal region. Thus, they were used for revealing tagSNPs in the gene regions studied (the threshold of squared correlation between SNPs r2 ≥ 0.8). For both tools, we estimated the tagging effectiveness (TE) as the ratio of the number of tagSNPs to the number of SNPs they tagged. 

Because of large number of potential tagSNPs and taking into account that not all of them could mark functionally important SNPs, the subsequent step was to annotate all the possible tagSNPs from high-LD regions with expression quantitative trait loci (eQTLs). For each gene, we downloaded the Significant Single-Tissue eQTLs using the web-interface of Genotype-Tissue Expression (GTEx) project (Release V8) [30]. The eQTLs were further intersected with the tagSNPs determined with Tagger algorithm and filtered by tissue defined as Brain, Artery, Nerve, Blood, and Heart. 

At the final step the tagSNPs from the Haploview’s Tagger runs with the maximal capture efficiency (maximal mean r2) and defying as eQTLs were selected to form a list of markers for studying in case–control associations using an appropriate genotyping approach (e.g., TaqMan real-time PCR assay).

The scripts used in this research are freely available at the repository https://github.com/inzilico/tagSNP (accessed on 9 August 2020). 

## 3. Results

**Figure 2:**
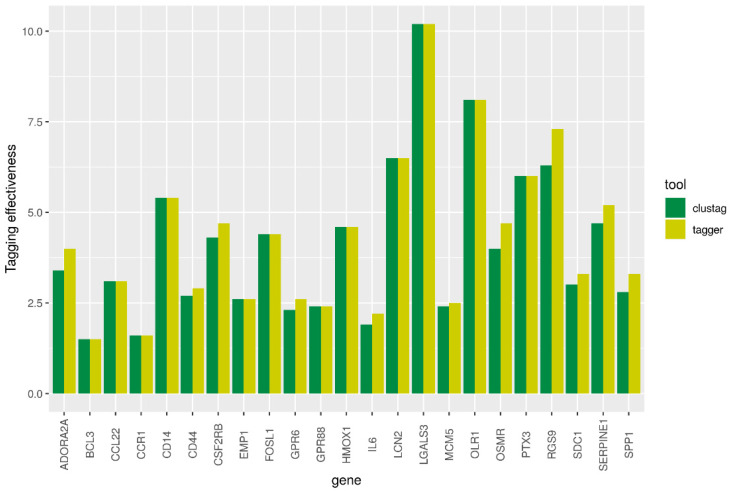
Tagging effectiveness by CLUSTAG and Tagger tools.

We extracted 23 of 24 human orthologues in rat using such projects as Ensembl, PANTHER, PhylomeDB, and MetaPhOrs. Different repositories resulted in the same list of orthologues that showed a one-to-one relationship between human and rat genes. The exception was Glycam1 gene, which orthologue was not identified. The human *GLYCAM1* is pseudogene. The genes extracted from Ensembl are presented in Table 1. The numbers of SNPs identified in each gene including flanking regions are given in Supplementary Table S1. The high-LD regions revealed with three approaches were in good agreement. The TE for CLUSTAG and Tagger are presented in Figure 2. In general Tagger demonstrated higher values of TE than CLUSTAG. Therefore, the tagSNPs revealed by Tagger were used for further analyses, particularly, searching eQTLs.

**Figure 3:**
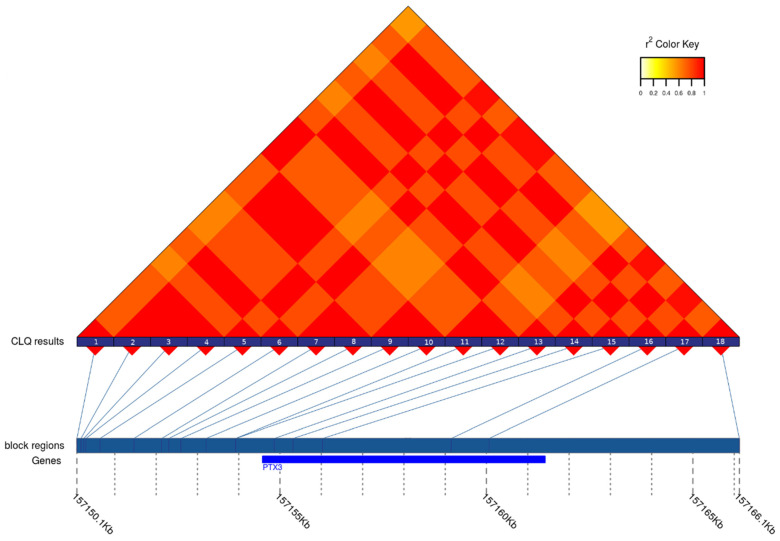
The heatmap of LD between the SNPs in the region of PTX3 gene in the CEU population. The numbers at the bottom of LD plot designate the SNPs included in analyses. Their coordinates as well as the boundaries of the gene are presented at the line below LD plot. The SNPs with numbers 2, 3, 4, 6, 9, 10, 13, 15, and 17 are the members of the first group of strongly associated (r^2^ ≥ 0.8) SNPs, while the SNPs with the numbers 5,7,8,14,16,18 and 1,11,12 represent the second and the third group, respectively.

Figure 3 represents the patterns of LD and tagSNPs revealed in PTX3 gene. All the tagSNPs obtained are given in Supplementary Table S2. Only part of them was found to be eQTLs. Some of such tagSNPs was the eQTLs for several tissues. On other hand, no eQTLs were identified among tagSNPs located in *BCL3, CCL22, FOSL1, GLYCAM1, GPR6, HMOX1, IL6*, and *LCN2* genes. After checking the identified sets of eQTLs, nine tagSNPs were determined as potential candidates for further analysis in case–control study using real-time PCR with TaqMan probes. Eight of them were associated with the changes of expression in brain tissues and thus to be the first-priority markers. The ninth locus—the SNP in CCR1 gene—had the greatest absolute values of eQTL-related statistics, particularly, *p*-value and normalized effect size (10^−47^ and −0.40, respectively).

## 4. Discussion

In this paper we proposed a workflow to identify the genetic markers associated with the outcomes of ischemic stroke. It is based on candidate gene approach that requires a prior knowledge about the system under consideration. We hypothesized that such information, particularly, a list of gene-candidates, can be taken from the model studies of brain ischemia in rat. Namely, we took 24 genes exhibited substantial changes in their expression in brain rat after tMCAO and using the workflow proposed obtained a list of the SNPs (tagSNPs with eQTLs abilities) that can be potentially applied in case–control studies.

In the line of workflow, we additionally compared four different sources of human orthologues in rat and three different methods for identification of high-LD regions and selection of tagSNPs. Ensembl, PANTHER, PhylomeDB, and MetaPhOrs were chosen because of the best accuracy and call rate of orthologues inference [31]. They all revealed the same list of human orthologues in rat and thus anyone can be used for searching of orthologs. Nevertheless, human orthologues in rat was identified for each gene of interest and confirmed by four different resources.

To explore patterns of LD and identify tagSNPs we used CLUSTAG, Tagger, and gpart tools. These methods were chosen because they represent three different approaches to the problem of identifying groups of highly correlated SNPs. Although they all exploit the LD-based approach and MAF to split the list of SNPs into high-LD regions (blocks), their algorithms differ. Tagger is based on the analysis of single markers and multi-marker haplotypes, CLUSTAG—on the analysis of clusters, while gpart—on graph analysis. gpart can effectively identify LD blocks of different range but cannot tag SNPs. In terms of TE, Tagger outperformed CLUSTAG and thus its tagSNPs were used for further analysis. However, the number of tagSNPs computed was still high for practical usage, which is why we annotated the SNPs from high-LD regions with eQTLs and subset the appropriate tagSNPs manually. Because the expression of a particular gene can be potentially affected not only the loci located inside the gene (cis-eQTLs) but the loci lied outside the gene (trans-eQTLs) [32] the workflow may be extended with searching additional distant loci associated with the changes of expression of target genes, particularly, the genes in which no cis-eQTLs were identified.

Like other studies pointed to establish genomic landscape of complex traits, our approach is also based on exploration of data of different types (mRNA transcription, population genetic variations, eQTLs) [33,34]. However, it does not rely on GWAS data which are known to be not good in identifying real causative variants and genes as well [35] and thus it is initially more confident. Another characteristic of our approach is its higher genetic complexity due to use of whole genome sequence data allowing possibility for involvement of higher number of real (not imputed) genetic loci in analysis. It should be also noted that although the workflow was applied to SNPs with frequency higher than 10%, it can be used for selecting and testing SNPs with lower frequency (e.g., loci with 5% to 1% frequency). However, it will require increasing the size of human samples analyzed (i.e., population sample, case and control samples). The data of Genome aggregation database project [36] that includes sequencing data of 1000 Genomes Project and others can be used for creating of samples with appropriate size.

The limitation of the proposed approach is that it has not been experimentally validated in a cohort of patients. Nevertheless, we believe that the created workflow will help both in studying of genomics of individual variability in ischemic stroke outcomes and looking inside the black box of polygenicity in their control.

## Figures and Tables

**Table 1:** The human orthologues of rat genes identified with Ensembl.

Rat				Human				Metrics				
Gene	Chrom	Start (bp)	End (bp)	Gene	Chrom	Start (bp)	End (bp)	1	2	3	4	5
Adora2a	20	16449385	16466147	ADORA2A	22	24813847	24838328	82	82	100	87.44	1
Bcl3	1	81996116	82010351	BCL3	19	45250962	45263301	82	83	100	59.56	1
Ccl22	19	10668403	10675173	CCL22	16	57392684	57400102	65	65	100	53.85	1
Ccr1	8	132147929	132153481	CCR1	3	46243200	46249887	80	80	75	100	1
Cd14	18	29265353	29266946	CD14	5	140011313	140013286	62	63	75	100	1
Cd44	3	99339455	99426032	CD44	11	35160417	35253949	71	68	100	91.02	1
Csf2rb	7	119544873	119558539	CSF2RB	22	37309670	37336491	56	56	100	100	1
Emp1	4	233415324	233449254	EMP1	12	13349650	13369708	76	74	75	100	1
Fosl1	1	227755887	227764393	FOSL1	11	65659520	65668044	92	91	100	100	1
Glycam1	7	142951738	142953998	*								
Gpr6	20	47518790	47521561	GPR6	6	110299514	110301921	94	94	50	99.76	1
Gpr88	2	237334865	237339419	GPR88	1	101003693	101007574	95	95	50	100	1
Hmox1	19	25622556	25629372	HMOX1	22	35776354	35790207	80	80	50	100	1
Il6	4	3095536	3100112	IL6	7	22765503	22771621	40	40	0	100	0
Lcn2	3	16763059	16766466	LCN2	9	130911350	130915734	64	64	100	100	1
Lgals3	15	28094062	28106276	LGALS3	14	55590828	55612126	82	78	100	96.41	1
Mcm5	19	25637492	25681915	MCM5	22	35796056	35821423	47	97	50	99.53	0
Olr1	4	211883405	211905489	OLR1	12	10310902	10324737	66	49	75	100	0
Osmr	2	75851664	75892056	OSMR	5	38845960	38945698	56	57	100	99.6	1
Ptx3	2	177457263	177463073	PTX3	3	157154578	157161417	81	81	100	100	1
Rgs9	10	97225541	97298645	RGS9	17	63133549	63223821	91	90	75	67.67	1
Sdc1	6	43667444	43689898	SDC1	2	20400558	20425194	77	76	100	100	1
Serpine1	12	24653385	24663763	SERPINE1	7	100770370	100782547	81	81	100	100	1
Spp1	14	6653093	6658953	SPP1	4	88896819	88904562	63	62	100	66.28	1

1—%id. target rat gene identical to query gene; 2—%id. query gene identical to target Rat gene; 3—rat gene-order conservation score; 4—rat whole-genome alignment coverage; 5—rat orthology confidence [0 low, 1 high]; *Glycam1 has no orthologues in human.

## Supplementary Materials

The following are available online at https://www.mdpi.com/2073-4425/12/3/328/s1, Table S1: The number of SNPs identified in human genes, Table S2: The gene based list of tagSNPs including those being eQTLs.
